# Cancer modeling by Transgene Electroporation in Adult Zebrafish (TEAZ)

**DOI:** 10.1242/dmm.034561

**Published:** 2018-09-27

**Authors:** Scott J. Callahan, Stephanie Tepan, Yan M. Zhang, Helen Lindsay, Alexa Burger, Nathaniel R. Campbell, Isabella S. Kim, Travis J. Hollmann, Lorenz Studer, Christian Mosimann, Richard M. White

**Affiliations:** 1 Memorial Sloan Kettering Cancer Center, Cancer Biology and Genetics and Department of Medicine, New York, NY 10065, USA; 2 Memorial Sloan Kettering Cancer Center, Developmental Biology, New York, NY 10065, USA; 3 Memorial Sloan Kettering Cancer Center, Gerstner Graduate School of Biomedical Sciences, New York, NY 10065, USA; 4 Memorial Sloan Kettering Cancer Center, 2017 Summer Clinical Oncology Research Experience (SCORE) Program, New York, NY 10065, USA; 5 Hunter College, New York, NY 10065, USA; 6 Institute of Molecular Life Sciences, University of Zurich, Zurich 8057, Switzerland; 7 SIB Swiss Institute of Bioinformatics, University of Zurich, Zurich 8057, Switzerland; 8 Weill Cornell/Rockefeller/Sloan-Kettering Tri-Institutional MD-PhD Program, New York, NY 10065, USA; 9 Memorial Sloan Kettering Cancer Center, Pathology, New York, NY 10065, USA; 10 The Center for Stem Cell Biology, Sloan Kettering Institute, New York, NY 10065, USA; Developmental Biology Program, Sloan Kettering Institute, New York, NY 10065, USA; 11 Weill Cornell Medical College, New York, NY 10065, USA

**Keywords:** Cancer, Electroporation, Melanoma, Zebrafish, Metastasis

## Abstract

Transgenic animals are invaluable for modeling cancer genomics, but often require complex crosses of multiple germline alleles to obtain the desired combinations. Zebrafish models have advantages in that transgenes can be rapidly tested by mosaic expression, but typically lack spatial and temporal control of tumor onset, which limits their utility for the study of tumor progression and metastasis. To overcome these limitations, we have developed a method referred to as Transgene Electroporation in Adult Zebrafish (TEAZ). TEAZ can deliver DNA constructs with promoter elements of interest to drive fluorophores, oncogenes or CRISPR-Cas9-based mutagenic cassettes in specific cell types. Using TEAZ, we created a highly aggressive melanoma model via Cas9-mediated inactivation of Rb1 in the context of BRAF^V600E^ in spatially constrained melanocytes. Unlike prior models that take ∼4 months to develop, we found that TEAZ leads to tumor onset in ∼7 weeks, and these tumors develop in fully immunocompetent animals. As the resulting tumors initiated at highly defined locations, we could track their progression via fluorescence, and documented deep invasion into tissues and metastatic deposits. TEAZ can be deployed to other tissues and cell types, such as the heart, with the use of suitable transgenic promoters. The versatility of TEAZ makes it widely accessible for rapid modeling of somatic gene alterations and cancer progression at a scale not achievable in other *in vivo* systems.

## Abstract (precis)

**Summary:** Here, we developed Transgene Electroporation in Adult Zebrafish (TEAZ), a method that enables researchers to study cancer and metastasis by introducing DNA elements focally into somatic adult tissue.

## INTRODUCTION

The zebrafish has become an increasingly applied model in cancer biology at the interface of basic discovery and preclinical animal experimentation. The high fecundity and relatively simple husbandry of zebrafish enable large experimental series *in vivo*. Early cancer models in zebrafish were largely developed using mutagens, such as N-methyl-N'-nitro-N-nitrosoguanidine (MNNG) or 7,12-dimethylbenz (α) anthracene (DMBA), which were later supplanted by transgenic technologies (Beckwith et al., 2000; Pliss et al., 1982; Spitsbergen et al., 2000). The initial transgenic cancer models were developed by injecting one-cell zebrafish embryos with DNA constructs containing a promoter and oncogene. For example, T-cell ALL was modeled by transgene-driven expression of the *MYC* oncogene using the *rag2* promoter, and melanomas generated by expressing *BRAF^V600E^* under the *mitfa* promoter in a *tp53^−/−^* germline mutant background (Berghmans et al., 2005; Langenau et al., 2003; Patton et al., 2005).

Despite the documented power of transgenic tumor models for mechanism discovery and drug testing, current models have several significant drawbacks: (1) the majority of established models do not exhibit spatiotemporal control, such that the timing and anatomical location of tumor onset remains variable (Kaufman et al., 2016; Patton et al., 2005; White et al., 2011); (2) they generally do not enable the introduction of serial somatic oncogenic events for modeling second and third hit mutations after onset (Mione and Trede, 2010; White et al., 2013); and (3) discerning multifocal primary tumors versus true metastatic spread of a single tumor is challenging. These issues all impose significant limitations for investigating tumor progression and metastasis.

Transplantation-based methods address some of these issues: tumors can be dissected from a transgenic tumor-bearing animal or from patient-derived xenografts (PDXs), and then serially transplanted into recipient animals such as the *casper* recipient strain to allow detailed *in vivo* imaging (Fior et al., 2017; Heilmann et al., 2015; Hoffman, 2015; Siolas and Hannon, 2013; Tang et al., 2016; White et al., 2008; Zeng et al., 2017). Alternatively, stable cell lines can be generated from a transgenic animal, such as the ZMEL1 melanoma line, which can similarly be used for transplantation studies (Heilmann et al., 2015). Although these transplantation approaches allow for precise spatiotemporal control and are amenable to imaging of metastasis, the experiments often require immunosuppression of the recipients either through irradiation or genetic manipulation of immune cells (Tang et al., 2016), in addition to the initial generation of the suitable cell line. Recent work from the Langenau laboratory has shown that syngeneic fish can be used as transplant recipients, but these require that the tumors be developed in that particular genetic background, somewhat limiting their broad use across cancer (Blackburn et al., 2011). Furthermore, transplantation cancer models implant foreign tumors into inherently artificial microenvironments.

A variety of Cre/Lox-based approaches have been used in the zebrafish to control the cells that undergo initiation, including T-cell leukemia that can be controlled by mRNA injection (Langenau et al., 2005). A further modification uses CreERT2 drivers, such that both the cell type and timing of gene expression can be controlled (Hans et al., 2009), and inducible expression has also been achieved with heat shock Cre constructs (Hans et al., 2011). Despite these advances, there is still a paucity of verified Cre/Lox*-*based approaches to cancer in the zebrafish as the transgenic animals have proven time-consuming to create, and require complex breeding schemes to generate the final required genotypes.

Based on this, we wished to develop an approach that would enable introduction of oncogenic elements directly into adult somatic tissue in a spatiotemporally controlled manner. In this paper, we report oncogenesis via Transgene Electroporation in Adult Zebrafish (TEAZ), which models how tumors natively form in somatic tissues in a fully immunocompetent adult zebrafish. Electroporation applies electrical pulses to generate pores within the cell membrane, enabling extracellular biomolecules (including DNA) to enter the cell (Neumann et al., 1982; Wong and Neumann, 1982). Electroporation is widely used for stable introduction of DNA elements into cells in tissue culture and into chick and mouse embryos. Electroporation has occasionally been utilized in adult zebrafish, but these studies have been limited to cell tracking and transient morpholino knockdowns, and the technique has never been applied to cancer modeling (Hoegler et al., 2011; Münch et al., 2013; Rambabu et al., 2005; Thummel et al., 2011). Several studies in mice have harnessed electroporation to introduce transgenes into select adult tissues, including retina, muscle, brain and prostate (Maresch et al., 2016; Nomura et al., 2016; Swartz et al., 2001; Yarmush et al., 2014), and used it to model tumors such as pancreatic cancer (Jung et al., 2014; Maresch et al., 2016; Park et al., 2014). However, these approaches require surgery of the mice and can only be limited to a small number of animals at a time, limiting the number of subjects that can reasonably be studied in each experiment. Based on these prior observations and the very large cohorts we can generate, we reasoned that direct electroporation of oncogenic transgenic constructs into the zebrafish would be a straightforward, highly scalable approach to model tumor formation in cells of interest. Because electrodes and DNA solutions can be placed at defined locations, TEAZ allows for delivery of multiple transgenes specifically to the anatomical locations of interest. We find that TEAZ allows for the development of complex, aggressive melanomas driven by expression of oncogenic BRAF^V600E^ in concert with loss of the tumor suppressors *tp53* and *rb1*. These tumors are highly invasive and eventually metastasize to distant locations, unlike previous transgenic zebrafish melanoma models which do not generally metastasize (Patton et al., 2005). Given the wealth of functionally uncharted genetic lesions discovered from sequencing human tumors, TEAZ allows for testing of candidate mutations in a rapid, scalable *in vivo* system. More broadly, TEAZ can also be used to study somatic alteration of gene function in any adult tissue, which will have applications for diseases outside of cancer as well.

## RESULTS

### Introduction of genetic elements into adult zebrafish via electroporation

**Fig. 1.**
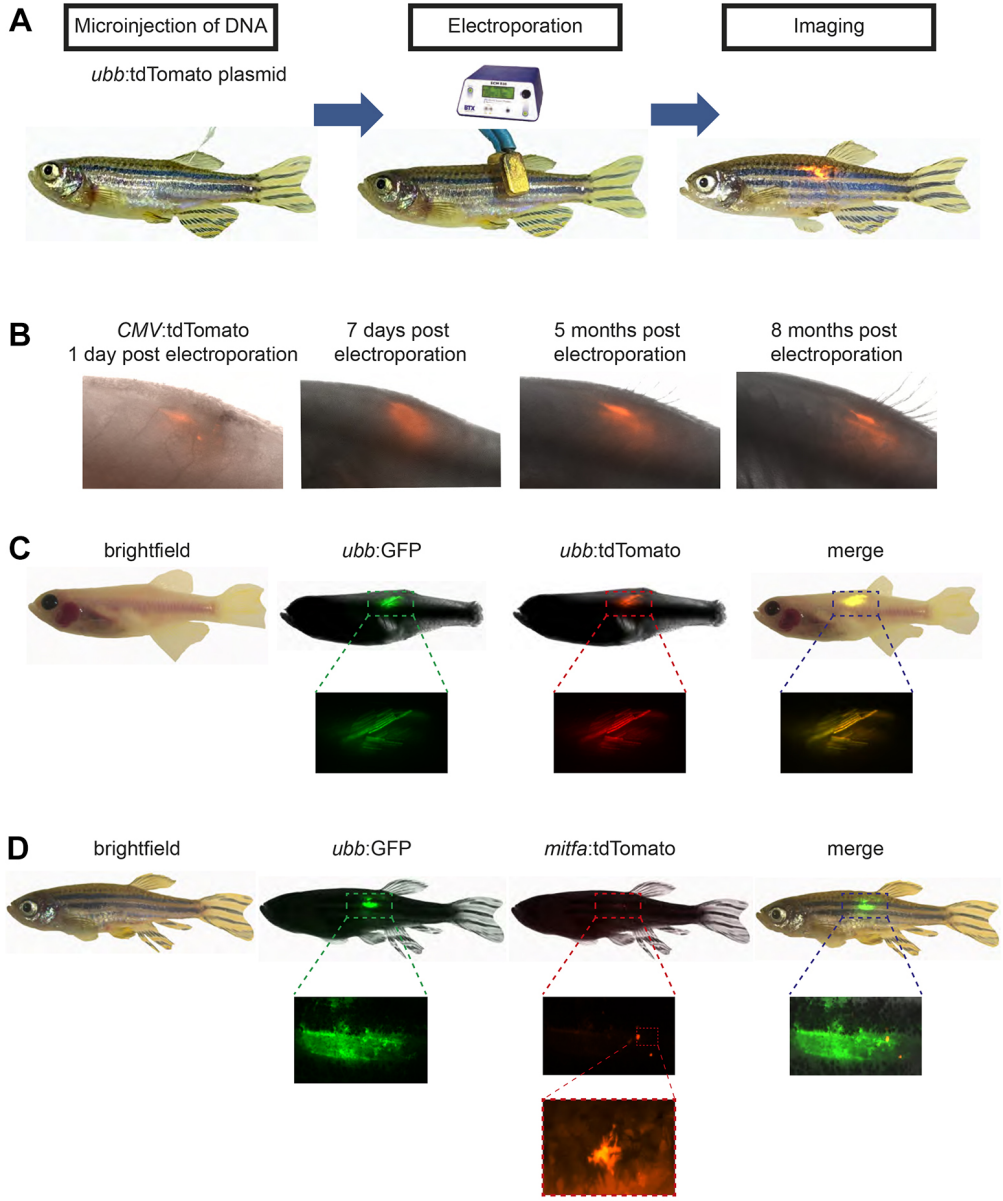
**TEAZ.** (A) Schematic representation of the method applied for the introduction of *ubb*:tdTomato directly under the dorsal fin of adult zebrafish. The purified plasmid DNA (1 μl of a 1000 ng/μl solution of *ubb*:tdTomato) is injected into anesthetized zebrafish using a pulled glass micropipette. Electrical pulses are directed across the injected region (settings: LV mode, 45 V, 5 pulses, 60 ms pulse length and 1 s pulse interval). Reporter expression can be visualized by fluorescent microscopy (*n*=2/2). (B) Electroporation of a *CMV*:tdTomato plasmid was performed and the animal followed for a period of 8 months (*n*=2/2). The fluorescent signal can be visualized as early as 1 dpe, with intensity peaking at ∼1 week and maintaining for at least 8 months. (C) Multiple plasmids will co-integrate in TEAZ. *casper* zebrafish were electroporated with a total volume of 1.0 μl (0.5 μl of 1000 ng/μl *ubb*:GFP and 0.5 μl of 1000 ng/μl *ubb*:tdTomato) and imaged using BF, GFP and tdTomato (*n*=3/3), revealing co-expression of the plasmids. (D) Promoter specificity is maintained following TEAZ. *AB* fish were electroporated with 1.0 μl total volume (0.5 μl of 1000 ng/μl *ubb*:GFP and 0.5 μl of 1000 ng/μl *mitfa*:tdTomato) and displayed highly restricted expression of the *mitfa* reporter plasmid, but widespread expression of the *ubb* plasmid (*n*=9/9). High-resolution imaging of the tdTomato^+^ cells reveals a dendritic phenotype, consistent with the melanocytic lineage.

The TEAZ method is designed to introduce genetic elements into specific locations within the adult zebrafish (Fig. 1A). The method has been optimized for the use of plasmids generated in *Escherichia* *coli* and purified using standard plasmid purification protocols (midi preps). To test this protocol, we anesthetized adult zebrafish, and then injected 1.0 μl purified plasmid DNA (1000 ng/μl) directly under the dorsal fin. The injected zebrafish was then quickly placed into an agarose mold to position the animal upright. Using paddle-shaped electrodes, we directed electrical pulses across the injected region (electroporator set to LV mode, 45 V, 5 pulses, 60 ms pulse length, 1 s pulse interval). To maximize expression in the skin as opposed to deeper tissues, the cathode paddle can be placed just above the surface where the DNA was injected as this will pull the negatively charged DNA towards the surface adjacent to the injection site. After electroporation, we placed the anesthetized zebrafish into fresh water for recovery and maintenance through standard husbandry. Including anesthetization, DNA injection and electroporation, the entire protocol takes ∼45-60 s per animal.

To establish TEAZ, we injected wild-type zebrafish (*AB* strain) with a plasmid in which the zebrafish *ubiquitin B* (*ubb*) promoter (Mosimann et al., 2011) drives tdTomato expression (*ubb*:tdTomato). This vector was created using the Tol2 transposon system that is commonly deployed in the zebrafish (Balciunas et al., 2006; Kawakami, 2007; Kawakami et al., 2000; Kwan et al., 2007; Villefranc et al., 2007). In our studies, we did not use transposase mRNA because in preliminary tests we were unable to drive fluorescence from mRNA (either GFP or tdTomato) following electroporation (data not shown). After TEAZ, we imaged the electroporated zebrafish starting at 1 day postelectroporation (dpe) and over the course of several months. Fig. 1B shows an example animal, in which stable expression of tdTomato (under the *CMV* promoter) was detectable for up to 8 months (Fig. 1B). Similarly, the stable expression of *ubb*:GFP-p2a-tdTomato was also detectable after 8 months postelectroporation (*n*=4/4). We also tested whether the same *ubb* promoter and TEAZ would express in other parts of the animal by injecting it directly into the head. We observed robust expression of *ubb*:GFP in the head in four of the five animals electroporated (Fig. S1), indicating that TEAZ-mediated transgene activity is not restricted to a particular location on the adult zebrafish body. At the site of electroporation, there is initially a small area of tissue damage, which is rapidly healed within 1 week. We have never seen ectopic expression of the transgene away from the site of electroporation, nor have we observed any systemic toxicity or obvious procedure-caused death in several hundred similarly electroporated animals. In addition, to ensure lack of germline transmission, we electroporated several different constructs (Table S1), waited for adult expression, and then bred those animals to wild-type adults. We then screened the resultant embryos at both 1 day postfertilization (dpf) and 4 dpf and saw no animals with fluorescence (*n*=947 embryos). This indicates that the TEAZ method allows for highly focal, somatic transgenesis at the site of electroporation.

### TEAZ allows for simultaneous expression of multiple plasmids

Electroporation of multiple plasmids into cultured cells *in vitro* generally leads to joint uptake of the plasmids by cells and subsequent co-expression of the transgenes. To test this in the zebrafish, we performed TEAZ with two plasmids in a single injection. We co-injected the *ubb*:tdTomato plasmid along with a *ubb*:GFP plasmid (each at 0.5 μl of 1000 ng/μl plasmid stock) and then monitored fluorescence. High-magnification views demonstrated that 100% of the transgene-expressing cells were double positive for both GFP and tdTomato (*n*=3/3 fish) (Fig. 1C). Consequently, TEAZ can be expanded to express and combine multiple transgenes in the adult zebrafish skin.

### Maintenance of promoter specificity following electroporation

We next sought to determine whether TEAZ enables cell type-specific transgene expression. We co-electroporated *mitfa*:tdTomato (Lister et al., 1999) (which drives in melanocytes) and *ubb*:GFP (which drives ubiquitously) plasmids and then imaged the zebrafish using both tdTomato and GFP channels. A representative animal is shown in Fig. 1D (*n*=9/9). As anticipated, we detected broad and strong expression of GFP from the *ubb* promoter (Mosimann et al., 2011). In contrast, we found highly limited expression of the *mitfa*:tdTomato plasmid. High-resolution imaging of the tdTomato^+^ cells revealed a dendritic appearance that is consistent with the appearance of mature melanocytes (Fig. 1D). We next tested expression in the heart using the cardiomyocyte-specific *myl7* promoter driving GFP (*myl7*:GFP; formerly referred to as *cmlc*:GFP) (Huang et al., 2003). We injected and electroporated *myl7*:GFP plasmid directly into the beating heart muscle of an anesthetized adult zebrafish. We found strong and specific expression of GFP in the beating heart (*n*=2/4) (Fig. S1C, Movie 1). Importantly, when *myl7*:GFP was electroporated below the dorsal fin (*n*=5) and *mitfa*:tdTomato was electroporated into the heart (*n*=5) fluorescence was not detected, showing that expression is highly cell type specific and driven by promoter specificity. We conclude that TEAZ-mediated vector delivery maintains promoter specificity following electroporation, enabling us to target specific somatic cell types within specified regions of adult zebrafish.

### Melanoma initiation requires multiple transgenes

We next sought to apply TEAZ to directly model melanoma formation in adult zebrafish, circumventing embryonic manipulations. We and others have previously used a traditional germline melanoma transgenic in which the *mitfa* promoter drives oncogenic BRAF^V600E^ (Patton et al., 2005; White et al., 2011). In a *tp53*^−/−^ deficient background, these transgenic animals develop a 100% penetrant melanoma at 4-12 months of age, without any additional transgenes (Patton et al., 2005; White et al., 2011). This original transgenic was further extended using the miniCoopR system (Ceol et al., 2011), in which the *mitfa* gene itself is knocked out, creating a strain with the genotype *mitfa:*BRAF^V600E^;*tp53*^−/−^;*mitfa*^−/−^ (herein referred to as the ‘triple’ strain). When this triple strain is injected at the one-cell embryo stage with a ‘rescue’ plasmid containing an *mitfa:mitfa* and *mitfa*:GFP cassette *in cis*, the resultant animals have rescued GFP^+^ melanocytes that all go on to develop GFP^+^ melanomas as adults (Ceol et al., 2011).

To test whether TEAZ is adaptable to this approach and could enable circumvention of initiating transgene expression at embryonic stages, we electroporated the miniCoopR:GFP rescue cassette under the dorsal fin of triple strain adult zebrafish. We found that 8/10 injected animals developed GFP fluorescence at the site of injection, and, remarkably, one of the animals developed rescued melanocytes. This indicates that it is possible to ‘rescue’ melanophore development in a germline genetic defect (i.e. *mitfa*^−/−^) by directly electroporating a minigene cassette into adult somatic tissues. However, none of these animals went on to develop melanoma over a period of 4 months, a duration that leads to melanoma in embryo injection-based experiments. This observation suggests that in TEAZ, additional genetic hits are necessary above and beyond BRAF and *tp53*^−/−^.

### TEAZ-mediated CRISPR of Rb1 stimulates melanoma in adults

**Fig. 2.**
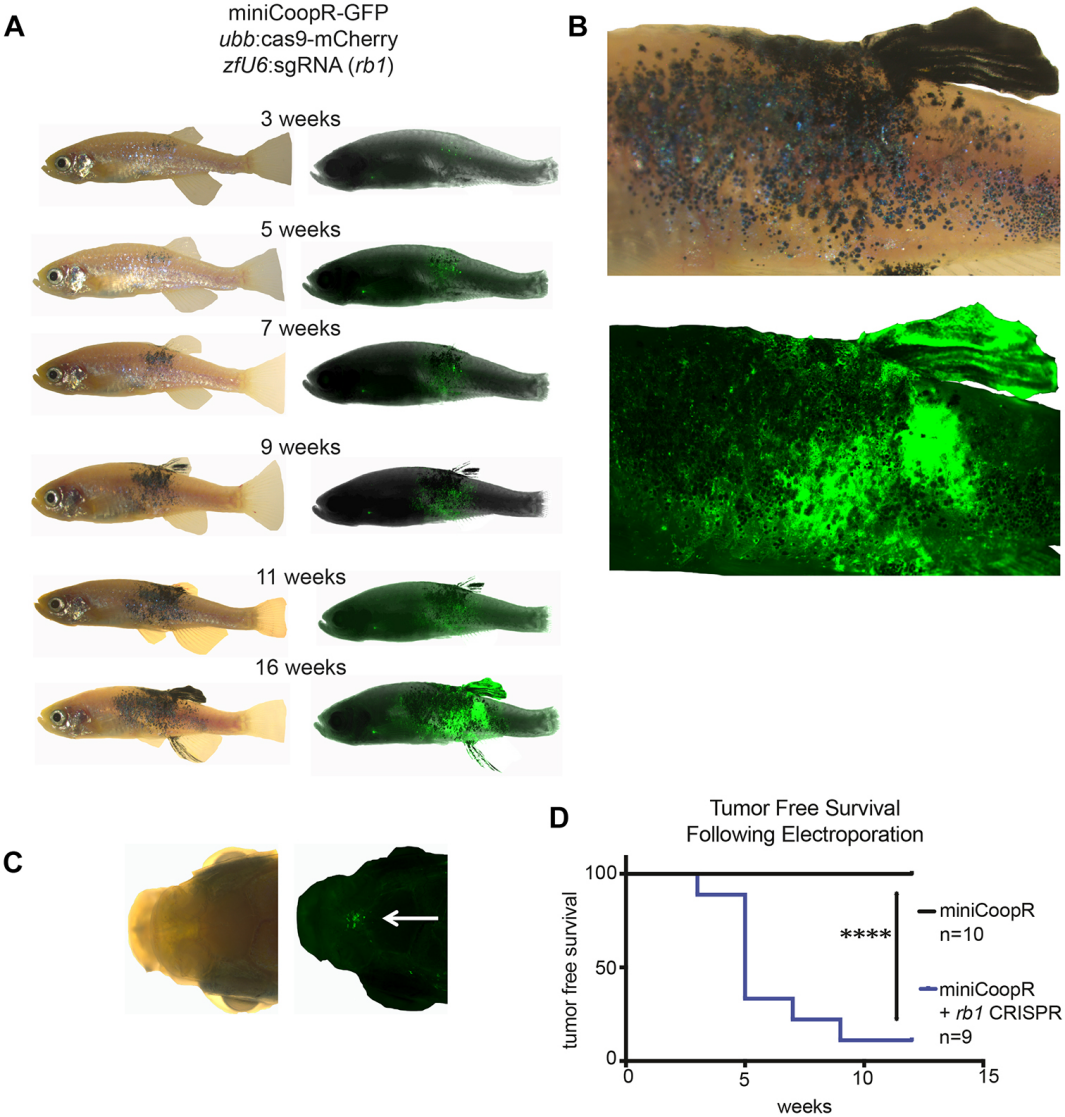
**Generation of a novel melanoma model with TEAZ.** (A) *mitfa:*BRAF^V600E^;*tp53^−/−^*;*mitfa^−/−^* zebrafish (triple strain) were electroporated with the miniCoopR:GFP plasmid that both rescues melanocytes and expresses GFP under the *mitfa* promoter, with (*n*=10) or without (*n*=9) two additional plasmids to genetically knockout *rb1* (*ubb*:Cas9 and *zfU6*:sgRNA against *rb1*). The electroporated zebrafish were then imaged over time by both fluorescence and brightfield to monitor tumor development. Overall, 17/20 electroporated zebrafish had GFP^+^ cells. Tumor development in a representative zebrafish from the melanoma model including *rb1* knockout is shown. (B) Higher-magnification view of the tumor-bearing animal shown in A at 16 weeks postelectroporation. (C) At 9 weeks postelectroporation, 4/8 zebrafish had evidence of GFP^+^ distant micrometastases in the head. (D) The loss of *rb1* is essential for tumor initiation as visualized by the Kaplan–Meier curve comparing zebrafish electroporated with miniCoopR:GFP with or without *rb1* sgRNA. Log-rank (Mantel–Cox) test was used for statistical analysis (****P<0.0001).

In the *mitfa*^−/−^ mutant background, there are no mature melanocytes (Lister et al., 1999) because *mitfa* is required for expression of melanocytic genes, such as the Pmel genes and *tyr*. We suspected that there might be melanocytic precursor cells in the *mitfa:*BRAF^V600E^;*tp53*^−/−^;*mitfa*^−/−^ background that are largely quiescent and not actively cycling. We therefore aimed to knock out the function of the tumor suppressor Rb1 using CRISPR-Cas9, because *TP53* and *RB1* mutations have a tendency to be concurrent in human melanoma patients, as seen in the cBIOPortal (*P*=0.017) (Berger et al., 2012; Cerami et al., 2012; Gao et al., 2013; Hodis et al., 2012; Hugo et al., 2016; Krauthammer et al., 2012). Rb1 normally acts to keep cells arrested in G1, and we therefore reasoned that loss of its function might provoke the cells to proceed through the cell cycle and be more amenable to full malignant transformation (Yu et al., 2009). To test this hypothesis, we employed the triple strain, and electroporated three plasmids: (1) miniCoopR:GFP, (2) *ubb*:Cas9 and (3) *zU6*:sgRNA against *rb1* (see Materials and Methods for details). We found that of the nine electroporated animals, eight developed rescued melanocytes and went on to establish aggressively growing GFP^+^ lesions with the phenotypic appearance of frank melanomas (Fig. 2A-C). The tumors appeared within 3-7 weeks, in striking contrast to the 3-6 months typically required for standard embryo-injection transgenics (Fig. 2D). To confirm that the effect was due to introduced *rb1* mutations, we dissected the dorsal fin (tumor) and tail fin (control normal tissue) from the same adult zebrafish for sequence analysis. Deep sequencing of the two fins and CrispRVariants-based allele analysis validated that the tumor contained two independent frameshift mutations in *rb1* at the protospacer adjacent motif site that are characteristic of CRISPR mutations and were not present in the control tail fin (Fig. S2) (Burger et al., 2016; Lindsay et al., 2016). Although the percentage of mutant reads was low in this analysis, it is likely because we sequenced surrounding normal tissue as well as tumor. We previously performed whole-genome and exome sequencing on a series (*n*=53) of embryonic-transgenic zebrafish tumors and did not see any Rb1 mutations (Kansler et al., 2017; Yen et al., 2013). Consistent with the loss of Rb1 in TEAZ tumors, when we stained for phospho-Rb1 in both a TEAZ tumor and standard embryo injection F0 tumor (i.e. *mitfa*:BRAF injected into a *tp53*^−/−^ background without any Cas9/sgRNA), we found that most of the cells in the TEAZ tumor are phospho-Rb1 negative, whereas most of the embryo injection tumor cells are Rb1^+^ (Fig. S3). The immunohistochemistry detected phospho-Rb1 but not total Rb1 protein, as we do not have an antibody that effectively stains for this. The results revealed that TEAZ-mediated transformation of adult tissue can result in rapid melanoma onset using tumor-relevant genetic lesions.

**Fig. 3.**
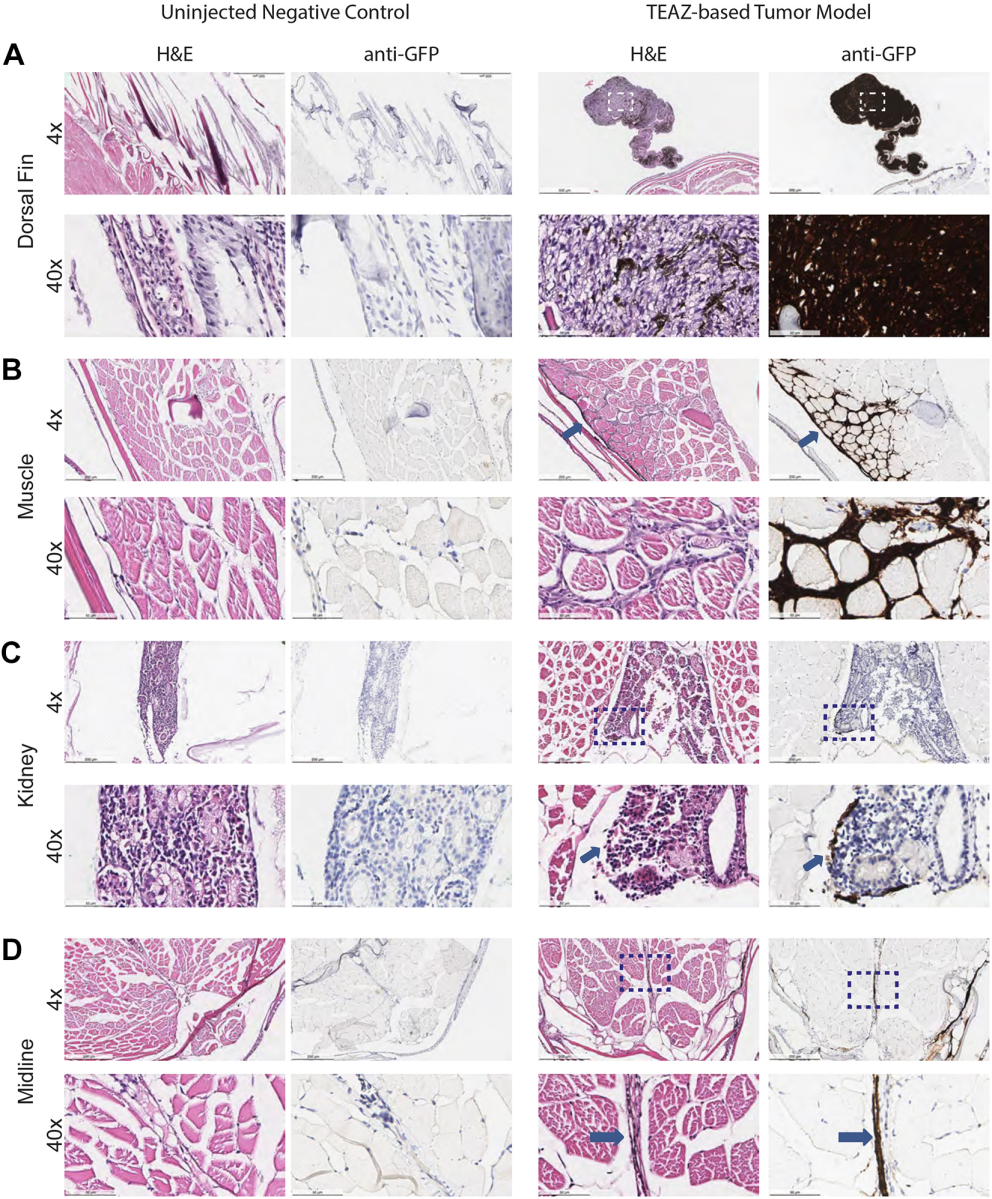
**Melanoma model using TEAZ show evidence of rapid progression.** (A) Pathology of tumor-bearing zebrafish (*n*=1) (along with control zebrafish, *n*=2) at 16 weeks postelectroporation, stained with H&E or anti-GFP immunohistochemistry, demonstrates a large primary tumor that is uniformly GFP^+^. (B-D) Histology reveals evidence of extensive invasion into the muscle (arrows) (B) along with micrometastatic sites within the kidney (C) or along blood vessels (arrows) (D). Images are visualized at 4× and 40×. Scale bars: 500 μm (4×) and 50 μm (40×). Dashed line boxes indicate the area enlarged at 40×.

To confirm that the lesions were truly tumorigenic, we followed a cohort of these TEAZ-treated zebrafish for a period of 4 months. By 5 weeks, primary melanomas could be visualized by both fluorescence and brightfield imaging. By 9 weeks, four of eight remaining zebrafish had tumors that had rapidly progressed, and traversed the midline to the opposite side of the body. In four of eight zebrafish, we noted evidence of GFP^+^ distant micrometastases in the head (Fig. 2C). To investigate further, we sacrificed two tumor-bearing animals along with two control animals and performed routine histology and anti-GFP staining. The first fish had a large protruding primary tumor with uniform GFP expression by fluorescence imaging. Histology of this tumor confirmed this, identifying uniform anti-GFP staining, and Hematoxylin and Eosin (H&E) staining showed cells highly consistent with high-grade melanoma, as determined by pathologist assessment (i.e. nuclear atypia and presence of melanin) (Fig. 3A). We noted extensive invasion into the muscle (Fig. 3B), which had not previously been seen in transgenic zebrafish melanoma modeling using BRAF^V600E^ with *tp53*^−/−^. Along with this invasion phenotype, we identified micrometastases within the kidney (Fig. 3C) and attached to blood vessels (Fig. 3D). The second fish had an atypical tumor with variable GFP expression by fluorescence imaging, and histology showed a tumor of mixed histology in the vicinity of the injection needle: surface GFP^+^ tumor cells consistent with a low-grade melanoma, and a deeper tumor in the muscle that was GFP^–^, consistent with a sarcoma (Fig. S4A,B). This second nonmelanoma tumor is likely caused by inactivation of both *rb1* and *tp53* in the muscle, as we used ubiquitous Cas9 in our studies, and this combination is commonly found in sarcomas (Cerami et al., 2012; Gao et al., 2013; Gonin-Laurent et al., 2007; Pérot et al., 2010; Stratton et al., 1990). We did not find any GFP staining or abnormal H&E staining in either of the control animals (Fig. 3; Fig. S4C). Taken together, our findings demonstrate that the TEAZ method can rapidly and robustly give rise to tumors in a highly defined spatiotemporal manner.

**Fig. 4.**
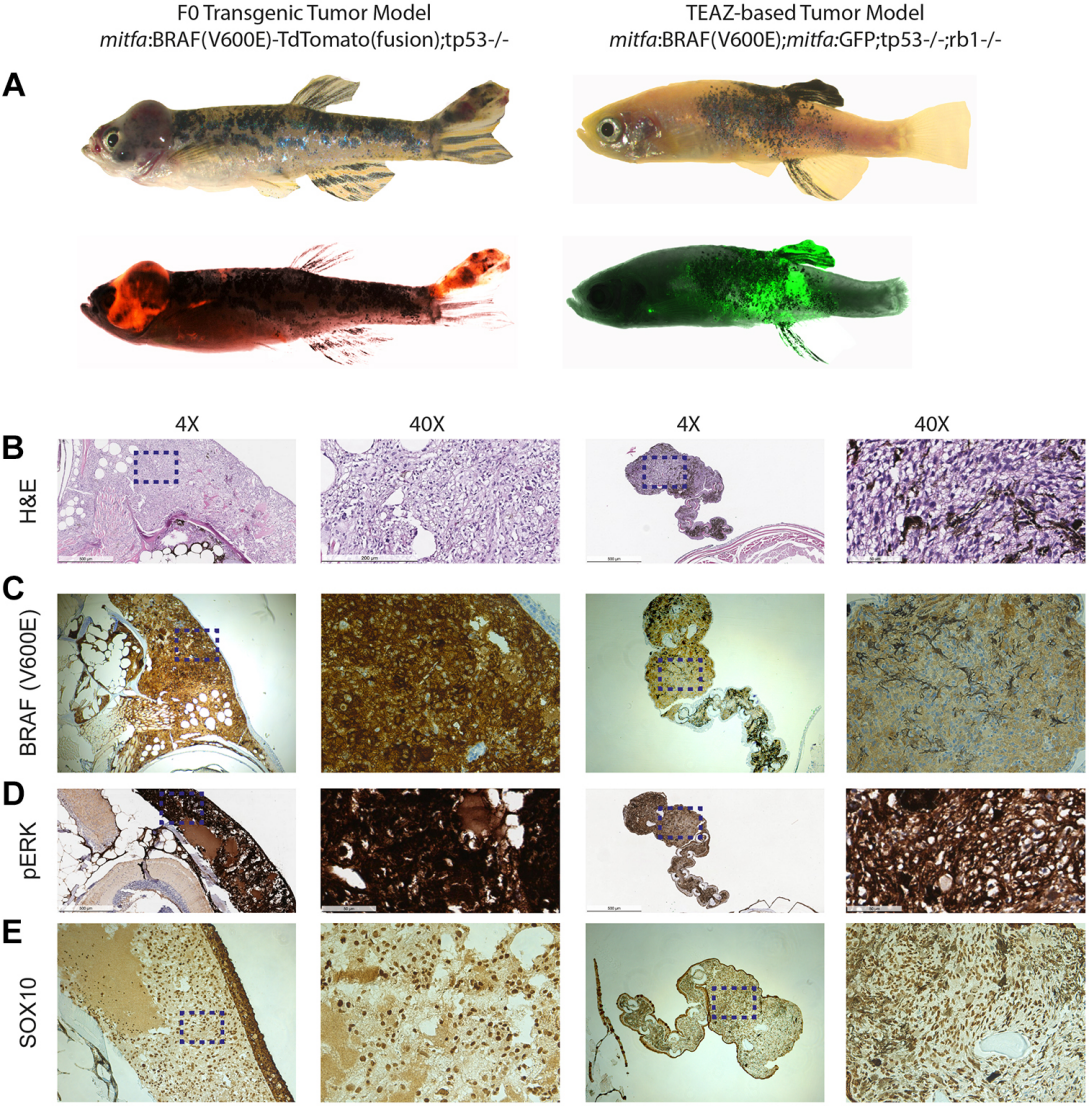
**Histological comparison of the embryo injection melanoma model and TEAZ melanoma model.** (A) The left images show a melanoma created by injection of an *mitfa*:BRAF^V600E^-tdTomato (fusion) transgene into a *tp53*^−/−^ background (*n*=1). Right images show a TEAZ-based melanoma created by electroporation of miniCoopR:GFP plus *ubb*:Cas9 plus *zfU6*:sgRNA against Rb1 (*n*=1) (example shown is fish at 16 weeks also shown in Fig. 2A). (B) H&E staining of both tumors shows similar histology, although with increased melanin pigmentation in the TEAZ tumor (also shown in Fig. 3A). (C,D) Antibody staining against BRAF^V600E^ shows that both tumors are widely BRAF^V600E^ positive, which correlates with high levels of phospho-ERK staining. (E) Reflecting the neural crest origin of melanocytes, both tumors show strong nuclear expression of SOX10. Images are visualized at 4× and 40×. Scale bars: 500 μm (4×) and 50 μm (40×). Dashed line boxes indicate the area enlarged at 40×.

We previously showed that most melanomas, of both fish and human origin, overexpress a neural crest transcriptional program typified by *crestin* and *sox10*, making the latter gene particularly relevant for human melanoma. To compare our TEAZ-based tumors to the more traditional embryo injection models, we performed immunohistochemistry of the melanomas using antibodies against SOX10, BRAF^V600E^ and phospho-ERK (Fig. 4). In agreement with previous models, we find that both the TEAZ melanomas and embryo injection transgenics have high levels of SOX10 protein expression. Additionally, both tumor types ubiquitously express both BRAF^V600E^ and phospho-ERK, albeit to a lesser degree in the TEAZ tumor. These data suggest that the TEAZ melanomas are functionally similar to the embryo injection transgenics. One key difference is that we also document evidence of progression and distant metastasis using TEAZ.

### Somatic tumors are amenable to sequential transgenic manipulation

**Fig. 5.**
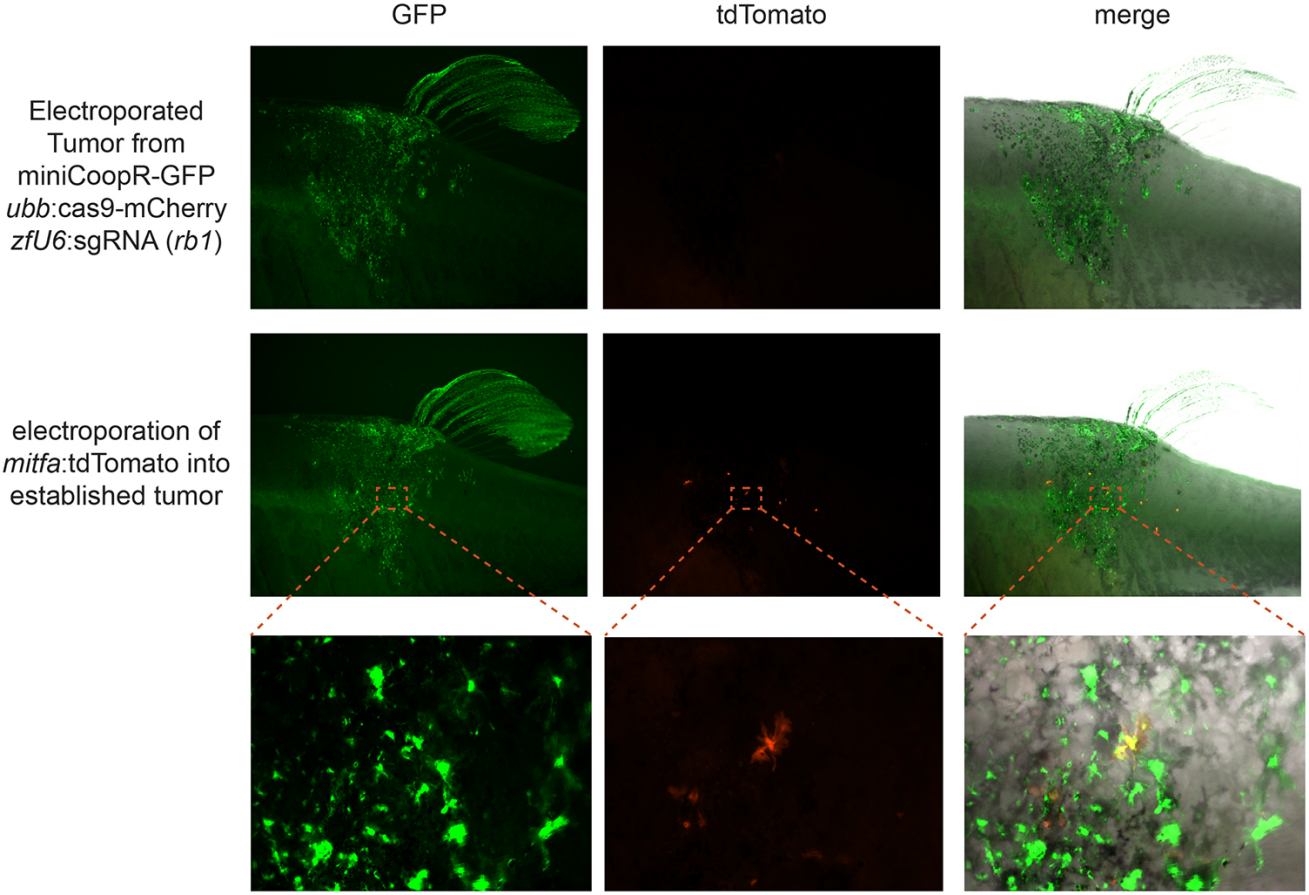
**Cancer modeling with TEAZ enables sequential electroporation of transgenes.** A tumor-bearing fish (created with *rb1* sgRNA as in Fig. 2) was imaged using GFP and tdTomato. As expected, only GFP^+^ tumor cells were seen, with no expression in the tdTomato channel. This tumor was then electroporated with an *mitfa*:tdTomato plasmid and re-imaged 5 days later, showing areas that are now both GFP^+^ and tdTomato^+^ (*n*=2).

One of the major limitations of available genetic models is the inability to modify genes in a sequential order, mimicking *in vivo* tumor progression from malignant clones (McGranahan and Swanton, 2017). This limitation precludes the investigation into whether certain oncogenic events are driving initiation (which occur early) versus metastasis (when they may occur later). We therefore sought to determine whether we could sequentially perform TEAZ to introduce new DNA elements into an already existing tumor. We selected a TEAZ melanoma from the cohort above (4 months postinitial electroporation), and electroporated an *mitfa*:tdTomato plasmid directly into the tumor (Fig. 5). Within 1 week after this second electroporation, we identified tdTomato^+^ cells within the TEAZ-treated tumor (*n*=2/2). The tdTomato^+^ cells have a dendritic appearance typical of a melanocytic cell. We also tested whether this sequential transgene electroporation into an existing tumor was of different efficiency to *de novo* electroporation into unperturbed tissue. To do this, we compared electroporation of *mitfa*:tdTomato into an existing TEAZ tumor versus electroporation of *mitfa*:tdTomato plus *ubb*:GFP into an *AB* fish (the GFP was added here to control for the fact that TEAZ tumors are GFP^+^). We then counted the number of tdTomato^+^ cells in both situations, and noted a greater number of positive cells when electroporating into the *AB* fish compared with the established tumor (Fig. S5). However, although it does appear that sequential electroporation into tumors might be slightly less efficient than *de novo* electroporation, it is still efficient enough for routine use in existing tumors and will allow for sequential modeling of genetic lesions.

## DISCUSSION

We have developed TEAZ, an electroporation-based approach for expressing transgenes and creating mutations in somatic cell types of interest within a region of interest in the adult zebrafish. We successfully applied TEAZ to the generation of malignant melanoma, and our results show that TEAZ can be used for sequential electroporation, which could be used to make increasingly complex tumor models. This model is amenable to initiating a tumor at a defined time and place, and will allow for a detailed analysis of tumor progression and metastasis in a fully immunocompetent zebrafish.

One major limitation of current transgenic cancer modeling in the zebrafish is the challenge of controlling both the timing and location of tumor initiation. Although both transplantation and Cre/Lox approaches can address some of these issues, neither fully solves the problem. Transplantation generally requires immune modulation of the recipient, and introduces relatively high cell burdens into tissue contexts that would not occur during natural tumor formation. Cre/Lox is extremely powerful but requires multiple genetic crosses, and there are very few verified lines that exist for cancer modeling in the fish. In contrast, TEAZ rapidly allows for introduction of the required genetic elements in a multiplexed, complex manner.

Electroporation has been used as a mechanism for tumor initiation in mouse models of cancer. Both glioblastoma and oligoastrocytomas (Chen et al., 2016) have been induced into the developing fetus using *in utero* electroporation and the piggyBAC transposon system. In these studies, the transposase was included on the plasmid as a helper, although in our study we did not find Tol2 transposase to be necessary for highly stable transgene expression. Recent work (Jung et al., 2014; Maresch et al., 2016; Park et al., 2014) showed that plasmid delivery and electroporation could be used to initiate KRAS-driven pancreatic cancer in the adult mouse and, similar to our findings, can be complemented with CRISPR-Cas9-mediated genome editing. One exciting area that we believe TEAZ will open up is the possibility of new epithelial cancer models in the zebrafish, because that has not been developed on a very large scale.

One of the major advantages of our TEAZ system is the high efficiency we have observed. Once trained, we find that the rate of success of transgene expression approaches 100% of injected animals with 100% survival when performed in the dorsal skin. Injections into other adult tissues, such as the heart or head, are technically more challenging, requiring more practice, and also result in higher death rates, as expected (75% survival for heart, 62.5% survival for head). In our study of the melanomas, we found that, overall, 88% of the fish developed GFP^+^ cells by 3 weeks, and all of those fish subsequently went on to develop tumors by 7 weeks. This timing is an advance over the previous standard transgenic melanoma models, even with the more rapid miniCoopR mosaic approaches that speed up tumors with the addition of oncogenes such as *SETDB1* (Chen et al., 2016). In addition, embryo injection remains a relatively laborious process, whereas we find that the adult electroporation is simple and fast and can be easily taught to inexperienced users.

One possibility that our TEAZ system opens up in the future is the ability to initiate adult-stage tumors in virtually any genetic background (i.e. the *casper* strain) or other existing transgenic line. In our studies using the miniCoopR background, we needed at least three genes to obtain efficient tumors: BRAF^V600E^, *tp53*^−/−^ and *rb1*^−/−^. This might or might not be related to the specifics of the miniCoopR system as the melanocyte progenitors likely to have to re-enter the cell cycle. We also noted that our tumors formed faster than typical miniCoopR tumors, even with the addition of genes such as *SETDB1*. This could relate to the specifics of the TEAZ approach or could simply be due to the accelerating effect of *rb1* loss. It was surprising that the allelic fraction of *rb1* mutations was low in our MiSeq analysis, but this could be due to the fact that, in isolating genomic DNA for sequencing, we included large margins of surrounding normal tissue along with the tumor. Our staining for phospho-Rb1 is suggestive that the majority of the tumor cells are deficient for this tumor suppressor. The extent of *rb1* loss required for TEAZ tumors will need further analysis.

It will be important for future studies to establish the minimum number of genetic elements necessary to drive tumor formation in wild-type backgrounds, which will allow TEAZ to be applied to existing transgenic lines that label a variety of interesting cell types, such as T-cells (Dee et al., 2016), macrophages (Ellett et al., 2011) or endothelial cells (Lawson and Weinstein, 2002). Related to this, we have not yet attempted TEAZ on larval-age fish because it will require specialized, smaller electroporation paddles, but this could be useful for modeling tumors that occur in young adults. Previously, creating transgenic tumors in a strain of interest required time-consuming breeding and genotyping to obtain the final genotype of interest. In contrast, with TEAZ, one can electroporate a combination of oncogenes and sgRNAs against tumor suppressors into any given genetic background directly, saving months of breeding and unused animals. To ensure specificity of the tumor types, it will be important to use tissue-specific promoters to drive Cas9, to avoid mixed-histology tumors induced by ubiquitous Cas9 expression.

Metastasis and tumor progression have remained challenging to study using the zebrafish model; our results presented here suggest that TEAZ-mediated tumor modeling is amenable to studying metastasis in a high-throughput, immunocompetent model. Although transplantation-based approaches are powerful, they require immune system manipulation, such as irradiation or transplantation in genetically immunocompromised zebrafish to counteract rejection (Heilmann et al., 2015; Tang et al., 2016; White et al., 2008, 2013). In contrast, TEAZ allows tumor formation in fully immunocompetent animals. These features render the TEAZ model well positioned to (1) screen metastatic modulators to test rate, propensity and latency; (2) selectively alter genes within specific cell types within the tumor microenvironment; (3) image the interplay between tumor cells and specific microenvironmental cell-types using widely available transgenic lines; and (4) introduce serial mutations to study order of progression or induce competition studies within a tumor.

## MATERIALS AND METHODS

### Zebrafish husbandry

Zebrafish were bred and maintained in the Zuckerman fish facility, in temperature- (28°C), pH- (7.4) and salinity-controlled conditions. All fish were maintained on a 14 h on/10 h off light cycle. The animal protocols described in this paper were approved by the Memorial Sloan Kettering Cancer Center Institutional Animal Care and Use Committee (12-05-008).

### Zebrafish mutant lines

Transgenic lines used in these studies included wild-type (*AB*), *casper* (D'Agati et al., 2017; White et al., 2008) and the triple line (Ceol et al., 2011) (*mitfa:*BRAF^V600E^;*tp53*^−/−^;*mitfa*^−/−^) (provided by the Houvras laboratory at Weill Cornell Medical College, New York, USA). TEAZ was equally successful in both male and female zebrafish. TEAZ was performed on zebrafish ranging from 4 to 12 months postfertilization.

### Molecular biology

Purified plasmids were generated using the Gateway system and isolated from *E. coli* using the Qiagen HiSpeed Plasmid Maxi Kit. The miniCoopR vector was provided by the Houvras laboratory. The zebrafish *U6* promoter was cloned out of genomic DNA as previously described (Heilmann et al., 2015). The sgRNA against *rb1* was designed using the CHOPCHOP software and has the 20 bp sequence 5′-GGCTCAGTGAGTTTGAACGG-3′ (Labun et al., 2016; Montague et al., 2014). The *ubb* promoter was as described previously (Mosimann et al., 2011), and the Cas9-mCherry fusion plasmid was subcloned from Addgene plasmid number 78313 (Burger et al., 2016). All final plasmids were constructed using Gateway technology and the Tol2kit as previously described (Kawakami, 2007; Kwan et al., 2007).

Plasmids used in the study were as follows: *ubb*:GFP (Mosimann et al., 2011) and *ubb*:tdTomato (both in Tol2kit plasmid backbone #394), *zfU6*:Rb1gRNA (Heilmann et al., 2015), *mitfa*:tdTomato (Lister et al., 2001), *myl7*:GFP (Huang et al., 2003), miniCoopR (Ceol et al., 2011) and *ubb*:Cas9-mCherry fusion (Burger et al., 2016) (all in Tol2kit plasmid backbone #395).

### Electroporation of adult zebrafish

At the time of electroporation, recipient adult zebrafish were anesthetized in 0.2% Tricaine. The plasmid of interest was resuspended at 1000 ng/μl in ddH2O, and 1.0 μl was injected into the dorsal skin, head or heart (using a pulled glass micropipette). No transposase mRNA was used in these studies. We have successfully tested a range of concentrations from 400 ng/μl to 2000 ng/μl and from 0.5 μl to 2.0 μl. Following injection, the zebrafish were immediately placed upright in an agarose mold for ease of handling, and electrodes were placed on either side of the fish surrounding the injection site (Fig. 1A). The cathode paddle was generally placed on the same side as the injection to promote the DNA entering cells closer to the surface of the fish, but the cathode and anode can be swapped to promote integration into cells deeper within the animal. We used the ECM 830 Electro Square Porator from BTX Harvard Apparatus and the Genepaddles, 3×5 mm. For all experiments described, the LV mode was used with a voltage of 45 V, 5 pulses, 60 ms pulse length and 1 s pulse interval. The electroporated zebrafish were immediately returned to flowing fresh water after electroporation. Electroporated zebrafish were imaged within 4 dpe to ensure successful TEAZ, and then serially imaged for up to 8 months using brightfield and fluorescence imaging.

For electroporation of deeper tissues, such as heart or brain, the same glass electrode was used to inject the DNA but penetrated more deeply directly into those tissues. The electroporation paddles were positioned around those organs, but still kept on the surface of the animal with the same electrical settings.

For the Rb1 miniCoopR experiments, the following concentrations of plasmids were used: miniCoopR:GFP (370 ng), *ubb*:Cas9 (205 ng) and *zfU6*:sgRNA against *rb1* (285 ng).

### Imaging and image processing

Adult zebrafish were imaged using an upright Zeiss Discovery V16 equipped with a motorized stage, brightfield, and GFP and tdTomato filter sets. To acquire images, zebrafish were lightly anesthetized with 0.2% Tricaine. Images were acquired with the Zeiss Zen software v1, and the postimage processing was performed using Fiji (Schindelin et al., 2012).

### Histology

Selected zebrafish were fixed in 4% paraformaldehyde for 48 h at 4°C and then paraffin embedded. Fish were sectioned at 5 µM and placed on Apex Adhesive slides, baked at 60°C, and then stained with H&E or antibodies against GFP (Abcam, ab183734, 1:100), BRAF^V600E^ (Abcam, ab228461, 1:400), phospho-Rb1 (Cell Signaling Technology, 8516s, 1:400), phospho-ERK (Cell Signaling Technology, 4370, 1:100) or SOX10 (Cell Marque, 383A-76, 1:50). All histology was performed by Histowiz (http://www.histowiz.com), and staining was performed by Histowiz or the Hollmann Laboratory and reviewed by a pathologist (T.J.H.).

### Kaplan–Meier analysis

All animals were followed for up to 16 weeks and tumor-free survival was measured using the Kaplan–Meier method. The differences between the miniCoopR zebrafish with and without *rb1* knockout were analyzed using the log-rank statistics.

### MiSeq analysis

Reads were mapped to the zebrafish genome version *GRCHz10* using bwa version 0.7.13-r1126. Mutation quantification was performed using CrispRVariants version 1.7.4 (Lindsay et al., 2016). MiSeq reads can be accessed through the NCBI Sequence Read Archive (SRA) (accession code SRP147816).

## Supplementary Material

Supplementary information
