# Supplementary material for: Cancer modeling by Transgene Electroporation in Adult Zebrafish (TEAZ)
Source: Dis Model Mech. 2018 Sep 27;11(9):dmm034561. doi: 10.1242/dmm.034561 (PMC6177007; doi:10.1242/dmm.034561)
Supplement: Supplementary information [file dmm-11-034561-s1.pdf]

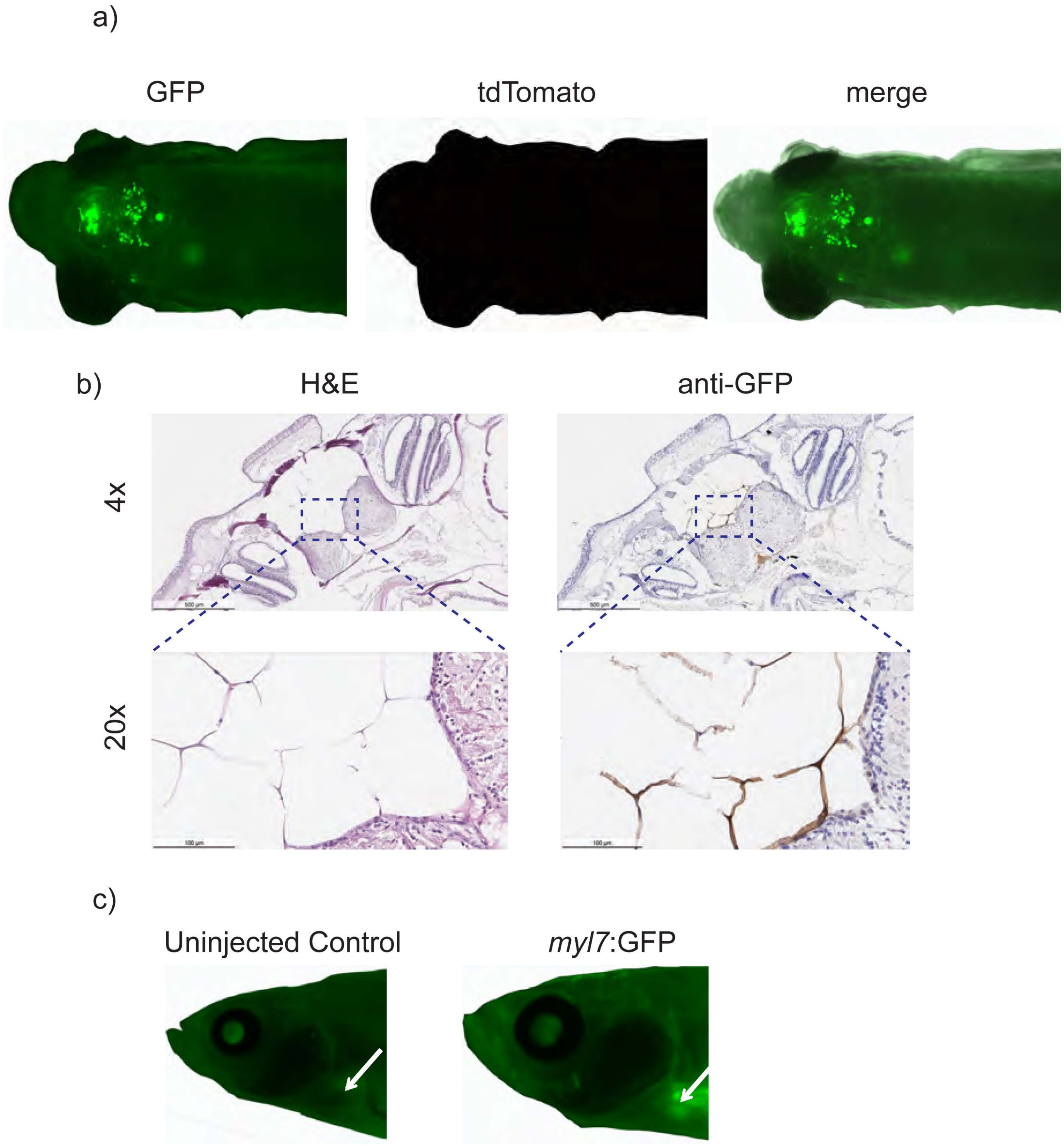

Disease Models & Mechanisms • Supplementary information

Fig. S1 TEAZ can be used to introduce transgenes in the adult brain and heart. (a) Purified plasmid encoding *ubb*:GFP (1µl of a 1000 ng/µl solution) was injected through the skull of an anesthetized *casper* zebrafish directly into the brain cavity using a pulled glass micropipette. The injected zebrafish is then electroporated across the dorsal-ventral axis of the head with the cathode positioned below the jaw and imaged for GFP fluorescence (n=4/5). (b) Pathology of the same electroporated *casper* zebrafish with hematoxylin and eosin or immunochemistry against GFP to demonstrate reporter expression. Images are visualized at 4x and 20x where scale bars represent 500 µm and 100 µm respectively. (c) TEAZ can be extended to expression within the heart of adult zebrafish. *Casper* fish were injected into the heart through the gills with 1 µl of 1000ng/µl of a plasmid carrying the *myl7*:GFP transgene (along with a *ubb*:Cre cassette that is unrelated for the purposes of this study) (n=2/4). Video of the beating fluorescent heart can be seen in Supplementary Video 1.

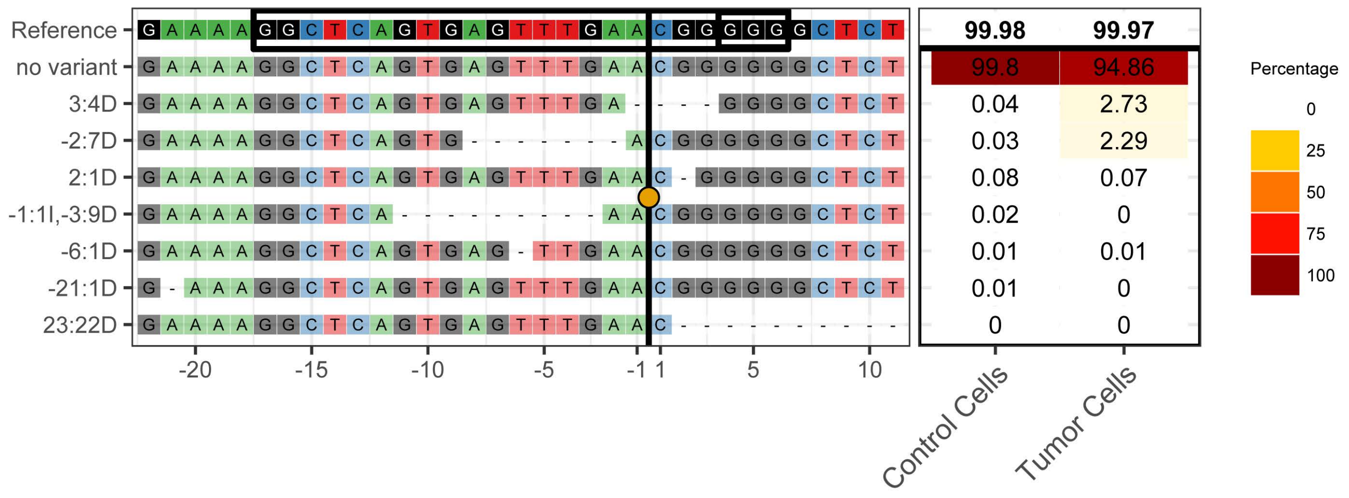

Fig. S2. A melanoma was induced as described in Main Figure 2, using *miniCoopR* and sgRNA against *rb1*. At 10 weeks post-electroporation, the melanoma was excised (n=1), along with control tissue from the tailfin of the same fish that was not electroporated. The tissues were digested for genomic DNA and the *rb1* locus was sequenced using multiplexed MiSEQ. In the tumor sample, we found a significant enrichment for two independent Cas9 induced mutations in *rb1* close to the *PAM* site (*3:4D* and *-2:7D*) at an allele fraction over 2% each. A small number of these reads (0.04%) were found in the control tissue likely due to barcode contamination during sequencing multiplexing(Ballenghien et al., 2017). Yellow circle represents an inserted cytosine in *-1:1I,-3:9D*.

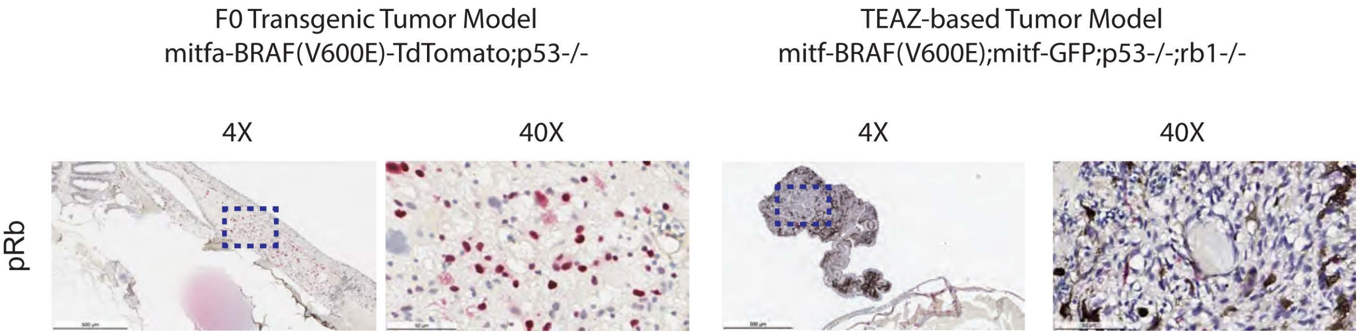

Fig. S3 Comparison of Rb1 expression in the F0 embryo injection melanoma (n=1) versus the TEAZ melanoma (n=1). On the left is a melanoma created by injection of an mitfa:BRAF-tdTomato(fusion) transgene into a p53-/- background. On the right is a TEAZ based melanoma created by electroporation of miniCoopR-GFP plus *ubb*:Cas9 plus *zfU6*:sgRNA against Rb1. Whereas most of the tumor in the traditional BRAF injected tumor is positive for Rb1 (red staining), the majority of the cells in the TEAZ tumor stain negatively.

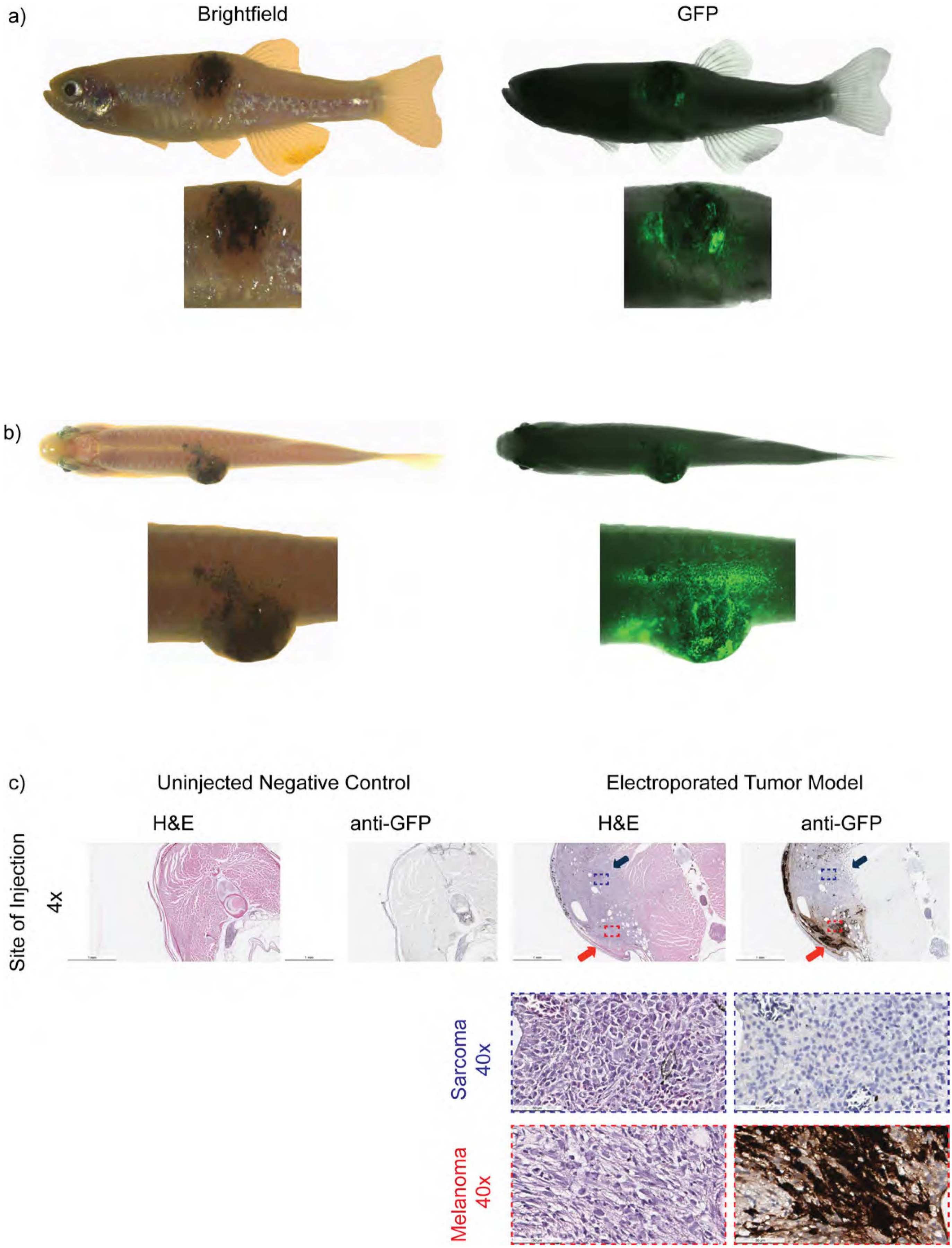

Disease Models & Mechanisms • Supplementary information

Fig. S4 Cancer model using TEAZ shows evidence of one tumor of mixed origin (n=1). (a) Fluorescent and brightfield imaging demonstrated a large primary tumor at the site of electroporation at 12 weeks post-electroporation with patchy GFP expression. (b) Imaging the zebrafish dorsally illustrates the large raised tumor from the contour of the animal. (c) Pathology of the tumor by hematoxylin and eosin or immunochemistry against GFP revealed that part of the deep tumor was GFP-negative and did not resemble a melanoma, but instead appeared consistent with a sarcoma. Images are visualized at 4x and 40x where scale bars represent 1 mm and 50 μm respectively. The blue boxes represent the area of sarcoma enlarged at 40x and the red boxes represent the area of melanoma enlarged at 40x.

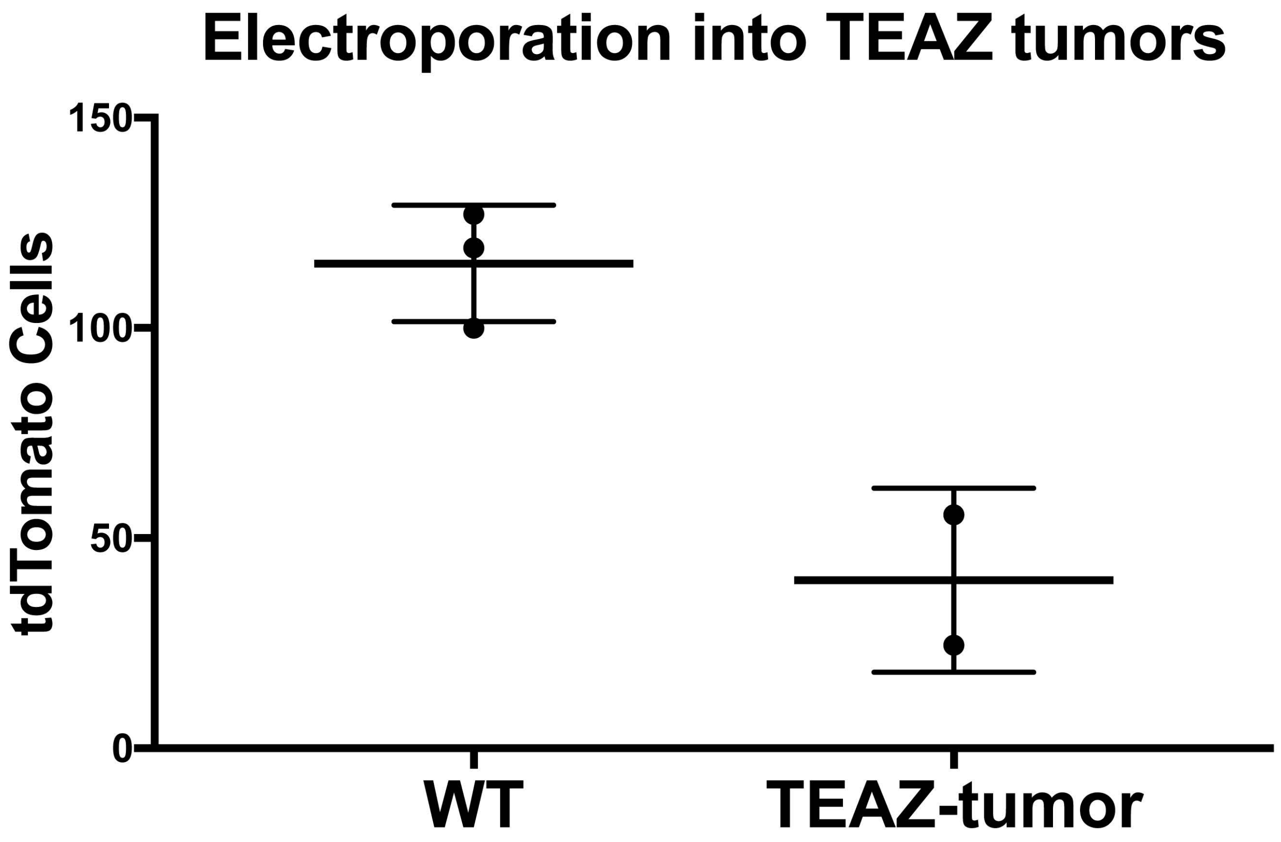

Fig. S5 Evaluation of electroporation efficiency of mitfa:TdTomato into TEAZ tumors for sequential transgene electroporation vs de novo electroporation into unperturbed tissue. We compared the number of red cells 4 days post electroporation of either *mitfa*:tdTomato into an existing TEAZ tumor (n=2) or electroporation of *mitfa*:tdTomato plus *ubb*:GFP into an AB fish (n=3) (the GFP was added here to control for the fact that TEAZ tumors are GFP positive).

Table S1. To ensure the absence of germline transmission following TEAZ, we electroporated several different constructs, waited for adult expression, and then bred those animals to WT adults. We then screened the resultant embryos at both 1dpf and 4dpf and we did not see any fluorescent offspring (n=947 embryos).

Absence of Germline Transmission with TEAZ

DNA Construct	Site of TEAZ	Days post TEAZ	Embryos 1DPF (fluorescent/total)	Embryos 4DPF (fluorescent/total)
p.mitf-TdTomato-sv40	Heart	70	0/18	0/18
p.ubi-TdTomato-sv40	Flank	140	0/521	0/521
p.ubi-GFP-sv40	Brain	182	0/195	0/195
p.sox10-GFP-sv40	Brain	189	0/213	0/213

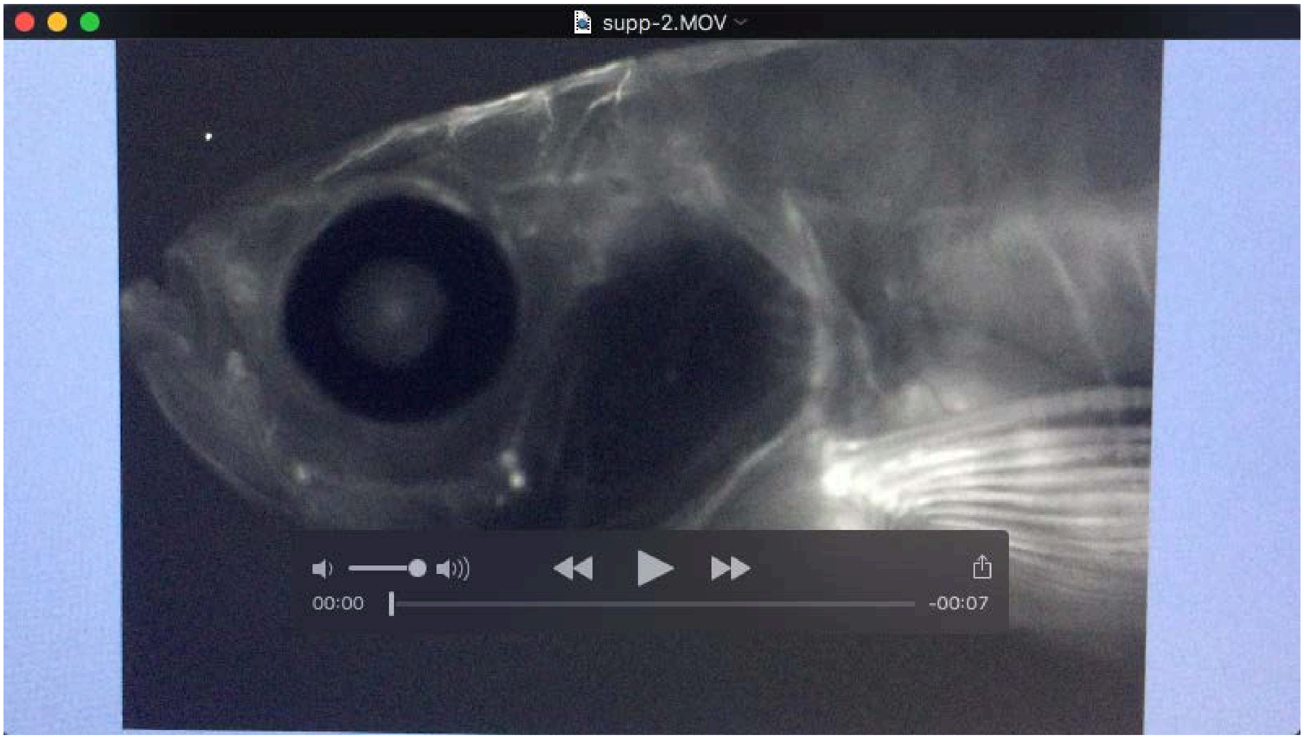

Supplemental Movie 1. TEAZ can be used to introduce transgenes in the adult heart. *Casper* fish were injected into the heart through the gills with 1 µl of 1000ng/µl of a plasmid carrying the *myl7*:GFP transgene (along with a *ubb*:Cre cassette that is unrelated for the purposes of this study) (n=2/4). Video of the beating fluorescent heart was taken in the GFP channel.
